# Biochemical Characteristics of Bone Mineral Metabolism before and throughout the First Year after Kidney Transplantation, Persistent Hyperparathyroidism, and Risk Factors in a Latin Population

**DOI:** 10.1155/2020/6913506

**Published:** 2020-03-10

**Authors:** Lourdes Balcázar-Hernández, Guadalupe Vargas-Ortega, Baldomero González-Virla, Martha Cruz-López, Raúl Rodríguez-Gómez, Ramón Espinoza-Pérez, Carlos Cuevas-García, Victoria Mendoza-Zubieta

**Affiliations:** ^1^Endocrinology Department, Hospital de Especialidades, Centro Médico Nacional Siglo XXI, Instituto Mexicano del Seguro Social, Cuauhtémoc 330, Colonia Doctores, 06720 México City, Mexico; ^2^Kidney Transplant Unit, Hospital de Especialidades, Centro Médico Nacional Siglo XXI, Instituto Mexicano del Seguro Social, Cuauhtémoc 330, Colonia Doctores, 06720 México City, Mexico

## Abstract

Bone mineral metabolism disease, which included persistent hyperparathyroidism, is common after successful kidney transplantation (KT) and is related with negative outcomes in kidney transplant recipients. There is a lack of information about bone mineral metabolism, persistent hyperparathyroidism, and its risk factors in Latin kidney transplant recipients (KTRs). *Material and Methods*: A retrospective study was conducted in 74 patients aged 18–50 years with evolution of 12 months after KT and estimated glomerular filtration rate (eGFR) >60 ml/min; biochemical data of bone mineral metabolism before and at 1, 3, 6, and 12 months of KT were registered. *Results.* Age was 33 (IQR 27–37) years; 54% (*n* = 40) were men. Before KT, all patients had hyperparathyroidism, 40% (*n* = 30) hypocalcemia, 86% (*n* = 64) hyperphosphatemia, and 42% (*n* = 31) hyperphosphatasemia. After KT, an increase of calcium and a diminution of PTH, phosphorus, and alkaline phosphatase were corroborated (*p*=0.001). All patients had hypovitaminosis D (deficiency: 91% (*n* = 67); insufficiency: 9% (*n* = 7)); 40% (*n* = 30) had persistent hyperparathyroidism at 12 months. Hyperphosphatasemia before KT (OR = 4.17 (95% CI: 1.21–14.44); *p*=0.04), hyperparathyroidism at 6 months (OR = 1.84 (95% CI; 1.67–2.06); *p*=0.02), hypovitaminosis D at 6 months (OR = 3.94 (95% CI: 1.86–17.9); *p*=0.01), and hyperphosphatasemia at 6 months (OR = 1.47 (95% CI: 1.07–2.86); *p*=0.03) were risk factors for persistent hyperparathyroidism at 12 months after KT. *Conclusion*. Persistent hyperparathyroidism at 6 months, hypovitaminosis D, and hyperphosphatasemia are risk factors for persistent hyperparathyroidism at 1 year of KT in Latin population.

## 1. Introduction

Disorders of mineral metabolism and bone disease are common in chronic kidney disease (CKD) patients, conditioning an increase of morbidity, diminution of quality of life, and increased cardiovascular mortality.The term “chronic kidney disease-mineral bone disorder” (CKD-MBD) has been used to describe a broader clinical syndrome that develops as a systemic disorder of mineral and bone metabolism due to CKD, which is manifested by abnormalities in bone and mineral metabolism and/or extraskeletal calcification. CKD-MBD includes three aspects: (1) laboratory abnormalities of calcium, phosphorus, parathormone (PTH), or vitamin D, (2) bone abnormalities in turnover, mineralization, volume, linear growth, or strength (osteodystrophy, osteopenia, osteoporosis, and low-mineral density), and (3) calcification of the vasculature or other soft tissues [1]. KT is the most effective treatment for end-stage renal disease. Many of complications of CKD may be reversed by transplantation; however, bone mineral metabolism disease (BMMD) may persist. Changes in mineral bone metabolism after KT have been described; however, there is a lack of information about bone mineral metabolism changes at long-term in Latin KTR. BMMD is common after KT and is influenced by factors such as preexisting renal osteodystrophy, immunosuppressive therapy, or kidney transplant dysfunction [1, 2]. Persistent hyperparathyroidism has been reported after KT, representing a related-factor with negative outcomes in KTR. The aims of our study were to describe the biochemical characteristics of bone mineral metabolism before and at 1, 3, 6, and 12 months after KT in Latin KTR and the frequency of persistent hyperparathyroidism at 1-year and its associated-factors.

## 2. Materials and Methods

### 2.1. Patients

We conducted a retrospective study at a tertiary care center. Eligible patients were men and women aged 18–50 years with 12 months of KT evolution, eGFR >60 ml/min, and no allograft dysfunction or rejection, enrolled in the clinic of bone mineral metabolism at Hospital de Especialidades, Centro Médico Nacional Siglo XXI in Mexico City.The biochemical evaluation of bone mineral metabolism involved the measurement of serum intact parathormone (PTH), serum 25-hydroxyvitamin D (25OHD), serum calcium, magnesium, phosphorus, albumin, alkaline phosphatase, 24-hour urinary calcium, and 24-hour urinary phosphorus; the biochemical data of bone mineral metabolism before TR and at 1, 3, 6, and 12 months of KT were registered for analysis. The eGFR was calculated by CKD-EPI. Hypovitaminosis D was established with concentrations of 25OHD below 30 ng/mL and classified under deficiency (below 20 ng/mL) or insufficiency (21–29 mg/mL); treatment was established according to the Endocrine Society's guidelines (cholecalciferol 6000–10,000 IU/d to obtain a 25OHD above 30 ng/ml, followed by maintenance therapy of 3000–6000 IU/d) [3]. Persistent hyperparathyroidism was defined as the presence of high concentrations of PTH at the first year despite the successful KT and 25OHD above 30 ng/mL. Tertiary hyperparathyroidism was diagnosed in KTR with persistent hyperparathyroidism and hypercalcemia and immunosuppression. Patients with living-donor KT received induction therapy with basiliximab, and patients with deceased donor KT received thymoglobulin. All patients had maintenance immunosuppressive therapy with corticosteroid (prednisone) associated with mycophenolate mofetil and tacrolimus.

### 2.2. Biochemical Measurements

For biochemical measurements, study participants underwent blood sampling after 8 h of fasting; urea (serum), creatinine (serum), calcium (serum and urine), magnesium (serum), phosphorus (serum and urine), albumin (serum), and alkaline phosphatase (serum) were measured by automated methods based on colorimetric and spectrophotometric assays (COBAS, Roche, EEUU). The normal ranges (NR) of these tests were as follows: serum calcium: 8.4–10.2 mg/dL; 24-hour urinary calcium: <300 mg/day; serum phosphorus: 2.7–4.5 mg/dL; 24-hour urinary phosphorus: 4–13 g/day; magnesium: 1.6–2.6 mg/dL; urea: 10–50 mg/dL; creatinine: 0.40–1.2 mg/dL; albumin: 3.5–5.2 g/dL; and alkaline phosphatase: males: 40–129 U/L; females: 35–104 U/L. The corrected serum calcium level (mmol/L) was calculated using the following formula: (4 g/dL-serum albumin concentration (g/dL)) × 0.8 + measured total serum calcium (mg/dL) with NR: 8.4–10.2 mg/dL. The urinary calcium-to-body weight ratio was the quotient of the division between 24-hour urinary calcium and weight in kg (NR <4 mg/kg body weight per day). A specific chemiluminescent assay was used to measure PTH (DiaSorin Inc, EEUU) with a sensitivity of 1 pg/ml and inter- and intraassay coefficient of variation (CV) of 5.3% and 3.5%, respectively, with a normal reference range of 15–65 pg/mL. Serum 25OHD levels were measured by the chemiluminescent method (DiaSorin Inc., EEUU) with a sensitivity of 4 ng/ml and inter- and intraassay CVs of 5.1% and 8.6%, respectively.

### 2.3. Statistical Analysis

The continuous variables were described as mean ± standard deviation (SD) or median and interquartile range (IQR) according to their distribution. For the categorical variables, proportions were used (expected frequency, prevalence). To establish the association between the continuous variables, the Student *t* test, Mann–Whitney test, or Wilcoxon signed-rank test were used, and for the categorical variables, the *χ*^2^ test was used. The Kruskal–Wallis test was performed to analyze the differences between groups. Correlations of quantitative variables were performed by Spearman's rank test. Multivariable logistic regression was used to identify risk factors for persistent hyperparathyroidism. All statistical tests were two-tailed; *p* < 0.05 was considered statistically significant. We used IBM SPSS Statistics V25.0 (IBM SPSS ®, EEUU) and STATA V14 (StataCorp ®, EEUU) as statistical software.

### 2.4. Ethical Approval

The study was conducted in accordance with the ethical principles specified in the Declaration of Helsinki, Good Clinical Practices, and regulatory requirements. Institutional scientific and ethics committees approved the study protocol. All patients provided written informed consent before participation.

## 3. Results

### 3.1. Clinical Characteristics

A total of 74 patients were included. Age was 33 (IQR 27–37) years. 54% (*n* = 40) were men. BMI was 24.6 (IQR 21.6–29). The most common etiology of primary nephropathy was congenital abnormalities of the kidney and urinary tract (CAKUT). 96% (*n* = 71) of patients had end-stage renal disease before KT, with dialysis as treatment. The time in dialysis before transplantation was of 29 (24–31) months. The 46% (*n* = 34) of patients received a related living-donor kidney transplant, 24% (*n* = 18) an unrelated living-donor kidney transplant, and 30% (*n* = 22) a deceased donor kidney transplant. Induction therapy for KT was performed with basiliximab in 70% (*n* = 52) and thymoglobulin in 30% (*n* = 22). Posttransplant immunosuppressive therapy was maintained with a dosage of prednisone of 20.0 (18.04–25.70) mg/day, mycophenolate of 1500 (1393–2128) mg/day, and tacrolimus of 6.0 (5.31–6.77) mg/day. The characteristics of KTR are summarized in Table 1.

### 3.2. Bone Mineral Metabolism before Kidney Transplantation

Before KT, all patients had secondary hyperparathyroidism; no patient had parathyroidectomy. Hypocalcemia was evidenced in 40% (*n* = 30) and hyperphosphatemia in 86% (*n* = 64); 42% (*n* = 31) had hyperphosphatasemia. No patient had hyper- or hypomagnesaemia, hypercalcemia, or hypophosphatemia. No patients received calcimimetics after KT. The biochemical characteristics of bone mineral metabolism are shown in Table 2.

### 3.3. Bone Mineral Metabolism after Kidney Transplantation

A PTH reduction of 87%, 91%, 85%, and 81% was evidenced at 1, 3, 6, and 12 months after KT, respectively. A statistical difference between PTH concentrations before KT and at 1, 3, 6, and 12 months, respectively, was corroborated (*p*=0.001). There no were differences in PTH between 1, 3, 6, and 12 months (*p*=0.39) (Table 2). An increase of calcium concentration was evidenced after KT (*p*=0.001) (Table 2). Hypercalcemia was found in 13% (*n* = 10) at 1 month, 9% (*n* = 7) at 3 months, 5% (*n* = 4) at 6 months, and 4% (*n* = 3) at 12 months. Hypocalcemia was present in 8% (*n* = 6) at 1 month and 1% (*n* = 1) at 3 months; no patient had hypocalcemia at 6 or 12 months. A diminution of phosphorus was found after KT (*p*=0.001) (Table 2); 31% (*n* = 23) had hypophosphatemia at 1 month, 34% (*n* = 25) at 3 months, and 5% (*n* = 4) at 6 months; no patient had hypophosphatemia at 12 months. An initial reduction of 25% in alkaline phosphatase was corroborated (*p*=0.001) (Table 2); hyperphosphatasemia was evidenced in 5% (*n* = 4) at 1 month, 7% (*n* = 5) at 3 months, and 1% (*n* = 1) at 6 months; no patient persisted with hyperphosphatasemia at 12 months. All KTR had hypovitaminosis D after KT; vitamin D measurement was performed only in 3 patients before KT. Deficiency was evidenced in 91% (*n* = 67) and insufficiency in 9% (*n* = 7) at 1 month; the time to achieve normalization of vitamin D was 18 (9–32) weeks, with a dose of 5000 (4000–6000) of cholecalciferol UI per day. The required dose of cholecalciferol for the maintenance of 25OHD above 30 ng/dL during the follow-up was 4000 UI/d (IQR 2000–4000). A statistical difference in vitamin D concentrations between 1, 3, 6, and 12 months after KT was evidenced (*p*=0.02) (Table 2). A diminution in magnesium was observed after KT (*p*=0.001); only 2 patients had hypomagnesaemia at the first month, without cases at 3, 6, and 12 months. Hyperphosphaturia was observed in 40% (*n* = 30) at 1 month, 31% (*n* = 23) at 3 months, 21% (*n* = 16) at 6 months, and 5% (*n* = 4) at 12 months. Hypercalciuria was evidenced only in one patient at 12 months. There were no statistical differences in serum calcium (*p*=0.52), phosphorus (*p*=0.59), 24 h urinary calcium (*p*=0.48), 24 hr urinary phosphorus (*p*=0.22), magnesium (*p*=0.78), urea (*p*=0.48), creatinine (*p*=0.28), or alkaline phosphatase (*p*=0.92) between 1, 3, 6, and 12 months after KT. There were no differences in bone mineral metabolism after KT according to gender, etiology of nephropathy, type of dialysis, or precedence of transplantation.

### 3.4. Persistent Hyperparathyroidism and Associated Factors

**Figure 1:**
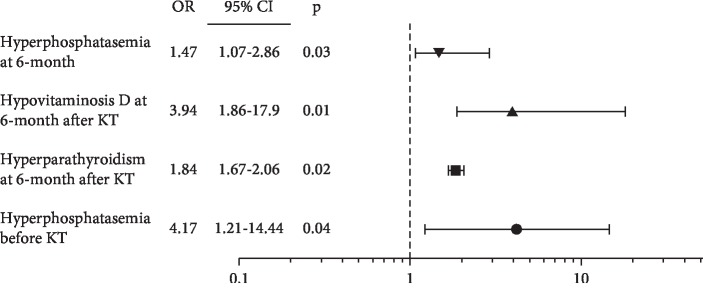
Risk factors for persistent hyperparathyroidism at 1 year after kidney transplantation.

Persistent hyperparathyroidism was found in 60% (*n* = 44) at 1 month, 50% (*n* = 37) at 3 months, 47.2% (*n* = 35) at 6 months, and 40% (*n* = 30) at 12 months; tertiary hyperparathyroidism was corroborated in 4% (*n* = 3), with the performance of subtotal parathyroidectomy and histopathological finding of adenoma plus parathyroid hyperplasia in all the cases; no parathyroidectomy was performed in patients without hypercalcemia. Patients with persistent hyperparathyroidism presented higher concentrations of PTH (699 (578–1218) vs 440 (66–851) pg/mL; *p*=0.04) and alkaline phosphatase (116 (66–250) vs 83 (68–128) U/L; *p*=0.008) before KT, as well as, higher PTH (143 (86–201) vs 42 (36–50) pg/mL; *p*=0.001) and alkaline phosphatase (116 (66–250) vs 83 (68–128) U/L; *p*=0.03) at 12 months after KT and lower vitamin D at 6 months (20.69 (17–32) vs 30.2 (28.2–40) ng/mL; *p*=0.04). There were no differences in other biochemical parameters of bone mineral metabolism (Table 3). The presence of persistent hyperparathyroidism at 12 months had a positive correlation with alkaline phosphatase before KT (*r* = 0.39; *p*=0.006), hyperphosphatasemia before KT (*r* = 0.34; *p*=0.02), vitamin D deficiency at 6 months (*r* = 0.39; *p*=0.007), hyperparathyroidism at 6 months (*r* = 0.60; *p*=0.001), PTH at 6 months (*r* = 0.86; *p*=0.001), and alkaline phosphatase at 6 months (*r* = 0.36; *p*=0.003), with a negative correlation with vitamin D concentrations at 6 months (*r* = −0.29; *p*=0.020). PTH concentrations at 12 months had a positive correlation with PTH before KT (*r* = 0.38; *p*=0.032), alkaline phosphatase before KT (*r* = 0.43; *p*=0.003), hyperphosphatasemia before KT (*r* = 0.32; *p*=0.026), vitamin D deficiency at 6 months (*r* = 0.39; *p*=0.007), hyperparathyroidism at 6 months (*r* = 0.60; *p*=0.001), and alkaline phosphatase at 6 months (*r* = 0.42; *p*=0.001), with a negative correlation with vitamin D concentration at 1 month (*r* = -0.45; *p*=0.002), and vitamin D concentration at 6 months (*r* = -0.48; *p*=0.001). Hyperphosphatasemia before KT (OR = 4.17 (95% CI 1.21–14.44); *p*=0.04), hyperparathyroidism at 6 months (OR = 1.84 (95% CI 1.67–2.06); *p*=0.02), vitamin D deficiency at 6 months (OR = 3.94 (95% CI 1.86–17.9)); *p*=0.01, and hyperphosphatasemia at 6 months (OR = 1.47 (95% CI 1.07–2.86); *p*=0.03) were risk factors for persistent hyperparathyroidism at 12 months after KT (Figure 1). A relationship between the doses of corticosteroid or cholecalciferol with PTH or persistent hyperparathyroidism at 12 months after KT was not evidenced.

## 4. Discussion

Several studies had reported the changes in bone mineral metabolism after KT; however, there is a lack of information of these modifications in Latin KTR. In this study, we evaluated the biochemical characteristics of bone mineral metabolism, before and throughout the first year after KT in a Latin population. Bone mineral metabolism presents different changes before and after KT; in patients with CKD, the declining kidney function results in decreased phosphorus excretion and reduced 1,25-dihydroxyvitamin D production, with a consequent reduction of serum calcium levels and increased PTH secretion. The elevation of FGF-23 in CKD is related with hyperphosphatemia and the perpetuation of the reduction of 1,25-dihydroxyvitamin D, which can aggravate the hyperparathyroidism in a vicious cycle. PTH and FGF-23 resistances could be found. KT is the most effective treatment for end-stage renal disease, with which the reversion of many complications of CKD is expected. After KT, circulating levels of FGF-23 and PTH are still high; however, a reduction of them resistance is evidenced. When the kidney function improves, a significant urinary phosphorus losses and hypophosphatemia are evidenced. PTH and FGF-23 decline towards normalization. The reductions in FGF-23 lead to a gradual increase in hydroxylation of 25OHD to active 1,25(OH)D, thereby leading to higher gut absorption of calcium and phosphorus, with a transitory hypercalcemia that precedes the normalization of calcium and phosphorus [1–4]. Hypovitaminosis D has been reported in more than 85% of KTR; the related causes include the following: (1) the reestablished function of 1*α*-hydroxylase (2) insufficient vitamin D supplementation before and after KT; (3) increased 25OHD catabolism induced by immunosuppressive drugs; (4) persistent FGF-23 hypersecretion after KT; and (5) the reduced sun exposure recommended to prevent skin cancers; hypovitaminosis D impacts in bone metabolism and immune system, having a relationship with dysfunction of kidney allograft, cardiovascular disease, osteoporosis, factures, cancer, and infectious diseases [5, 6]. Due to the different changes in bone mineral metabolism, the Kidney Disease Improving Global Outcomes (KDIGO) recommends the early and routine monitoring of calcium-parathyroid hormone-vitamin D in KTR [7]. In our series, we evidenced a progressive reduction of PTH and alkaline phosphatase during the first year after KT; hypercalcemia was observed during the first 3 months, with a low frequency at 1st year; hypophosphatemia showed predominance during the first 3 months, with remission at 1 year; hypovitaminosis was found in all KTRs, with a requirement of high doses of cholecalciferol and longer time for achieve normalization of 25OHD. The improvement of bone mineral metabolism after successful KT is expected; however, KTR has multiple associated factors of BMMD.

BMMD after KT is characterized by changes in bone quality and calcium-parathyroid hormone-vitamin D axis alterations and is related with a low quality of life and a high risk of fracture, morbidity, and mortality [8]. Preexisting renal osteodystrophy at time of KT, effects and consequences of transplantation-specific therapies on bone (immunosuppression), and the effects of reduced renal function after KT are the major associated factors [9]. Secondary and tertiary hyperparathyroidism, hypovitaminosis D, osteopenia, osteoporosis, osteomalacia, and pathologic fractures are included in BMMD [7–9].

After KT, PTH decreases, especially, during the first three months; high PTH concentrations after 3 months have been related with persistent hyperparathyroidism, that may or may not improve over time. At 6 months after KT, PTH remains above normal in approximately one-third of patients. Spontaneous resolution of persistent hyperparathyroidism occurs within the first year in approximately 50% of KTR; however, a 30% to 60% can persist after 1 year, even 21% after 15 years [4, 10, 11]. Persistent hyperparathyroidism after KT is associated with negative outcomes, including low bone density, fractures, vascular calcification, cardiovascular disease, nephrocalcinosis, allograft dysfunction, graft loss, and all-cause mortality [10, 11]. Long dialysis duration, high PTH prior to KT, high calcium or high alkaline phosphatase after KT, impaired kidney function after KT, parathyroid hyperplasia, older age, large maximum parathyroid gland size before KT, and monoclonal transformation (nodular hyperplasia) of parathyroid glands have been reported as risk factors of persistent hyperparathyroidism [9–11].

We found a similar frequency of persistent hyperparathyroidism at 1-year (40%) in Latin KTR, evidencing its association with high alkaline phosphatase before and after KT, hyperparathyroidism before and at 6 months after KT, and low concentrations of 25OHD. Hyperphosphatasemia before KT and at 6 months, hyperparathyroidism at 6 months, and vitamin D deficiency at 6 months were risk factors for persistent hyperparathyroidism in our population.

These findings lead a timely diagnosis and treatment of associated-factors to avoid the presence and the deleterious consequences of persistent hyperparathyroidism [2–4, 10, 11]. An important point to emphasize is the evidence that changes in bone mineral metabolism are independent of gender, etiology of nephropathy, type of dialysis, or precedence of KT; these data show that the assessment of bone status is essential in all KTRs.

The main strength of our study is the evaluation of biochemical parameters of bone mineral metabolism both before and after KT in Latin KTR, which represents the first published study that evaluates bone mineral metabolism at long term in this specific population. The limitations include the design of study and the lack of resources to complete bone mineral metabolism evaluation, including vitamin D status, before KT. We propose the establishment of strategies for prevention, diagnosis, and treatment of BMMD before and after KT, with the development of longitudinal studies in this population, to overcome these limitations.

## 5. Conclusion

Successful KT improves the biochemical parameters of bone mineral metabolism throughout the first year; however, persistent hyperparathyroidism can be found in KTR. Persistent hyperparathyroidism at 6 months after KT, hypovitaminosis D, and hyperphosphatasemia (before and after KT) are risk factors for persistent hyperparathyroidism at 1 year of KT.

## Figures and Tables

**Table 1:** Baseline characteristics of kidney transplant recipients included in the study.

Baseline characteristics of kidney transplant recipients	
Gender, % (*n* = )	Women: 46 (34)
	Men: 54 (40)

Age (years), M (IQR)	33 (27–37)

Ethnicity, (*n* = )	Latin (74)

BMI (kg/m^2^), M (IQR)	24.6 (21.6–29)

Chronic kidney disease etiology, % (*n* = )	CAKUT: 43 (32)
	Uncertain: 33 (24)
	Glomerulonephritis (membranous, proliferative, and focal segmental glomerulosclerosis): 8 (6)
	Autoimmune: 4 (3)
	Diabetic nephropathy: 8 (6)
	Hypertensive nephropathy: 4 (3)

KDIGO classification stage before kidney transplantation, % (*n*=)	Stage 4: 4 (3)
	Stage 5: 96 (71)

Dialysis before kidney transplantation, % (*n*=)	96 (71)

Type of dialysis before kidney transplantation, % (*n*=)	Only peritoneal dialysis: 46 (34)
	Only hemodialysis: 14 (10)
	Peritoneal dialysis before hemodialysis: 36 (27)

Type of kidney transplant, % (*n*=)	Related living-donor kidney transplant: 46 (34)
	Unrelated living-donor kidney transplant: 24 (18)
	Deceased donor kidney transplant: 30 (22)

CAKUT = congenital abnormalities of the kidney and urinary tract; M = median; IQR = interquartile range.

**Table 2:** Bone mineral metabolism characteristics before and at 1, 3, 6, and 12 months after kidney transplantation.

	Before KT	1 month after KT	3 months after KT	6 months after KT	12 months after KT
PTH (pg/mL)	654 (307–905)	79 (41–167)^*∗*^	57 (32–142)^*∗*^	96 (46–180)^*∗*^	118 (49–183)^*∗*^
Calcium (mg/dL)	8.9 (8.5–9.6)	9.4 (9.2–10)^*∗*^	9.9 (9.7–10.2)^*∗*^	10.2 (9.3–10.4)^*∗*^	10.3 (9.6–11)^*∗*^
Corrected calcium (mg/dL)	8.9 (8.3–9.8)	9.5 (8.9–9.8)^*∗*^	9.5 (9.2–10.4)^*∗*^	9.8 (9.7–10.5)^*∗*^	10.2 (10.2–11)^*∗*^
Phosphorus (mg/dL)	5.4 (4.1–7.2)	2.6 (1.5–4.1)^*∗*^	2.9 (1.8–3.6)^*∗*^	2.8 (2.2–3.1)^*∗*^	2.8 (2.7–3.1)^*∗*^
Magnesium (mg/dL)	2.5 ± 0.3	1.7 ± 0.2^*∗*^	1.6 ± 0.2^*∗*^	1.6 ± 0.2^*∗∗*^	1.7 ± 0.2^*∗*^
Albumin (g/dL)	4.3 (3.7–4.5)	4.5 (4.2–4.7)	4.8 (4.5–5)	4.5 (4.4–4.7)	4.7 (4.6–4.8)
Vitamin D (ng/mL)	Nonquantified	10.6 (6.9–26.8)	18.4 (9.2–35.7)^*∗∗*^	24.8 (16.4–36.11)^*∗∗*^	48 (31–49)^*∗∗*^
Alkaline phosphatase (U/L)	110 (81–147)	83 (61–207)^*∗*^	114 (72–206)	89 (68–188)^*∗*^	101 (90–110)^*∗*^
24-hour urinary calcium (mg/d)	—	70 (10–140)	80 (30–105)	60 (10–140)	70 (40–110)
Urinary calcium-to-body weight ratio (mg/kg/d)	—	1.1 (0.7–2.3)	1.3 (0.6–1.7)	1.0 (0.3–2.2)	1.1 (1–1.8)
24-hour urinary phosphorus (mg/d)	—	56 (43–69)	42 (35–47)	54 (26–90)	42 (41–63)
Urea (mg/dL)	123 (104–162)	46 (35–53)^*∗*^	41 (32–55)^*∗*^	34 (30–43)^*∗*^	31 (30–56)^*∗*^
Creatinine (mg/dL)	11.5 (8.4–14.8)	1.2 (0.9–1.5)^*∗*^	1.2 (0.9–1.4)^*∗*^	1.0 (0.9–1.2)^*∗*^	0.91 (0.9–1.3)^*∗*^
Estimated glomerular filtration rate: CKD-EPI (ml/min)	4.6 (3.9–6.5)	74 (60–86)^*∗*^	68 (62–89)^*∗*^	74 (67–81)^*∗*^	72 (62–83)^*∗*^

Data reported as mean ± SD or median and IQR; ^*∗*^*p*=0.001 vs pretransplant; ^*∗∗*^*p* < 0.05 between 1, 3, 6, and 12 months.

**Table 3:** Bone mineral metabolism characteristics according to the presence of persistent hyperparathyroidism at 12 months after kidney transplantation.

Bone mineral metabolism before and after kidney transplantation	Patients with persistent hyperparathyroidism	Patients without persistent hyperparathyroidism	*p*
*Bone mineral metabolism before kidney transplantation*			
PTH (pg/mL)	699 (578–1218)	440 (66–851)	0.04
Calcium (mg/dL)	134 (109–189)	101 (72–159)	0.48
Corrected calcium (mg/dL)	10 (9.3–10.3)	9.6 (9.3–10.1)	0.82
Phosphorus (mg/dL)	5.7 (4.3–7.6)	5.4 (3.9–8.2)	0.30
Magnesium (mg/dL)	2.6 (2.3–2.7)	2.6 (2.1-2-6)	0.35
Alkaline phosphatase (U/L)	116 (66–250)	83 (68–128)	0.008
Urea (mg/dL)	111 (101–155)	152 (73–168)	0.77
Creatinine (mg/dL)	11.7 (7.9–14)	11.3 (7.4–20)	0.16
Estimated glomerular filtration rate: CKD-EPI (mL/min)	4.6 (3–6.6)	4.9 (3.6–7)	

*Bone mineral metabolism after kidney transplantation*			
PTH (pg/mL)	143 (86–201)	42 (36–50)	0.001
Calcium (mg/dL)	10 (9.3–10.9)	10.2 (9.7–10.5)	0.59
Corrected calcium (mg/dL)	10 (9.3–10.3)	9.6 (9.3–10.1)	0.91
Phosphorus (mg/dL)	2.2 (1.8–3.1)	2.7 (2.3–3.6)	0.63
Magnesium (mg/dL)	1.7 (1.6–1.9)	1.8 (1.5–1.9)	0.40
Vitamin D (ng/mL)	20.69 (17–32)	30.2 (28.2–40)	0.04
Alkaline phosphatase (U/L)	116 (66–250)	83 (68–128)	0.03
24-hour urinary calcium (mg/d)	90 (62–137)	92 (75–110)	0.80
24-hour urinary phosphorus (mg/d)	50 (42–80)	44 (34–63)	0.74
Urea (mg/dL)	45 (31–56)	39 (31–56)	0.65
Creatinine (mg/dL)	1.1 (0.9–1.4)	1.3 (0.9–2.3)	0.76
Estimated glomerular filtration rate: CKD-EPI (ml/min)	72 (63–82)	70 (47–93)	0.35

Data reported as mean ± SD or median and IQR.

## Data Availability

All data generated or analyzed during this study are included in this published article. The database generated during the current study is available with the corresponding author on reasonable request.

## Abbreviations

KT: Kidney transplantation
KTR: Kidney transplant recipients
CKD: Chronic kidney disease
CKD-MBD: Chronic kidney disease-mineral bone disorder
BMMD: Bone mineral metabolism disease
eGFR: Estimated glomerular filtration rate
PTH: Parathormone
25OHD: Serum 25-hydroxyvitamin D
CAKUT: Congenital abnormalities of the kidney and urinary tract
KDIGO: Kidney disease improving global outcomes.
